# Clinical Trial Sites and Malaria Incidence Mismatch

**DOI:** 10.4269/ajtmh.25-0142

**Published:** 2025-05-13

**Authors:** Siddharth Brahmbhatt, Mukul Jain, Nilima Kshirsagar

**Affiliations:** Zydus Research Centre Ahmedabad, India E-mail: siddharth.brahmbhatt@zyduslife.com; Zydus Research Centre Ahmedabad, India E-mail: mukul.jain@zyduslife.com; Emeritus Professor, Maharashtra University of Health Sciences Nashik, India; Former National Chair, Indian Council of Medical Research Government of India New Delhi, India; Former Dean, Director, Medical Education and Health GSMC KEM Hospital Mumbai, India E-mail: kshirsagarna@yahoo.in

Dear Editor,

We read, with great interest, the January 2025 Supplemental Issue of the *AJTMH* (112-1), which highlights the importance of surveillance data for transformative malaria control and elimination.^1^ It quite correctly emphasizes the need for data-driven decision making. The focus is on high quality and comprehensive surveillance data on vectors and parasites, including molecular and genomic data and use of digital technology. The WHO global malaria program has also advocated subnational tailoring and provides guidance for using granular data. It is recommended that countries must strengthen their surveillance systems to detect early signs of partial artemisinin resistance, which could derail malaria control within a country.

Surveillance of antimalarial drug efficacy is needed for timely detection of, and response to, resistance. However, we wish to highlight gaps in the conduct of clinical trials that lead to challenges in the development of effective regional guidelines and policies, culminating in inefficient malaria-control programs. In a scoping review, Arena et al highlighted how nearly two-thirds of the malaria parasite-positive subjects presented to health facilities were excluded from antimalarial treatment trials, with the reasons for exclusion not reported for nearly one-third of these subjects.^2^ This gap in data is concerning, as the study populations may not be representative of their communities. In addition, studies have shown that genotypes vary across regions, for both the host and the parasite, thus the efficacy of drugs may vary. Therefore, pragmatic trials with appropriate geographic representation would improve insights into malaria control and elimination.

**Figure 1.**
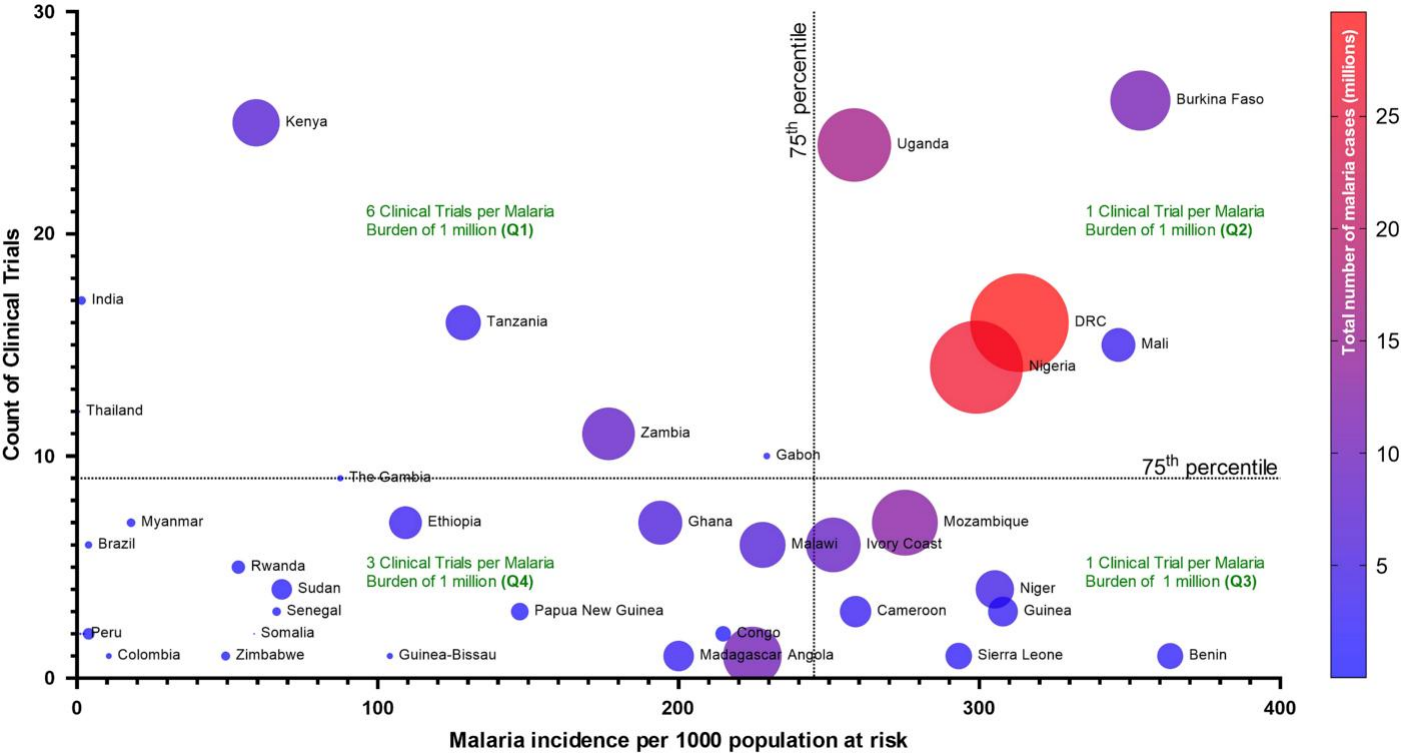
Count of clinical trials relative to malaria incidence. Bubble size and color represent total estimated number of malaria cases.

It is important that clinical studies are done in countries or regions with high malaria burdens. However, we have identified a mismatch between the malaria burden and the frequency of treatment efficacy trials. We obtained data for clinical trials, which are currently recruiting, from the WHO International Clinical Trials Registry Platform and extracted the number of national clinical trials.^3^ For multi-region trials, each country listed in the record was included. Further, we extracted data on malaria burden, i.e., estimated malaria incidence (per 1,000 population at risk) and total number of malaria cases (presumed + confirmed cases) from the WHO Global Health Observatory. A bubble scatter plot was then generated to assess the correlation between malaria burden and the number of clinical trials (Figure 1).^4^ The size of the bubble and color were adjusted based on the total estimated number of malaria cases.

This analysis points to a disproportionate intensity of clinical trials in countries that have relatively low malaria burdens. For instance, a subset of relatively low burden countries (Q1) are running an estimated six clinical trials per million malaria cases, while some high burden countries (Q2) are running only one clinical trial per million malaria cases (Figure 1). Further, among high-burden countries, drug efficacy trials remain restricted to only a few countries, in particular, the Democratic Republic of Congo, Uganda, Burkina Faso, Nigeria, and Mali. This mismatch between malaria burden and trial intensity may be limiting the abilities of many high-burden countries to best control malaria.
